# Directional selection coupled with kin selection favors the establishment of senescence

**DOI:** 10.1186/s12915-023-01716-w

**Published:** 2023-10-23

**Authors:** András Szilágyi, Tamás Czárán, Mauro Santos, Eörs Szathmáry

**Affiliations:** 1 Institute of Evolution, HUN-REN Centre for Ecological Research, Budapest, Hungary; 2 Departament de Genètica i de Microbiologia, Grup de Genòmica, Bioinformàtica i Biologia Evolutiva (GBBE), Universitat Autònoma de Barcelona, Barcelona, Spain; 3 cE3c – Centre for Ecology, Evolution and Environmental Changes & CHANGE – Global Change and Sustainability Institute, Lisbon, Portugal; 4 Center for the Conceptual Foundations of Science, Parmenides Foundation, Pöcking, Germany; 5 Department of Plant Systematics, Ecology and Theoretical Biology, Eötvös Loránd University, Budapest, Hungary; 6 Konrad Lorenz Institute for Evolution and Cognition Research, Klosterneuburg, Austria

**Keywords:** Aging, Evolution, Longevity, Evolvability, Kin selection

## Abstract

**Background:**

Conventional wisdom in evolutionary theory considers aging as a non-selected byproduct of natural selection. Based on this, conviction aging was regarded as an inevitable phenomenon. It was also thought that in the wild organisms tend to die from diseases, predation and other accidents before they could reach the time when senescence takes its course. Evidence has accumulated, however, that aging is not inevitable and there are organisms that show negative aging even. Furthermore, old age does play a role in the deaths of many different organisms in the wild also. The hypothesis of programmed aging posits that a limited lifespan can evolve as an adaptation (i.e., positively selected for) in its own right, partly because it can enhance evolvability by eliminating “outdated” genotypes. A major shortcoming of this idea is that non-aging sexual individuals that fail to pay the demographic cost of aging would be able to steal good genes by recombination from aging ones.

**Results:**

Here, we show by a spatially explicit, individual-based simulation model that aging can positively be selected for if a sufficient degree of kin selection complements directional selection. Under such conditions, senescence enhances evolvability because the rate of aging and the rate of recombination play complementary roles. The selected aging rate is highest at zero recombination (clonal reproduction). In our model, increasing extrinsic mortality favors evolved aging by making up free space, thereby decreasing competition and increasing drift, even when selection is stabilizing and the level of aging is set by mutation-selection balance. Importantly, higher extrinsic mortality is not a substitute for evolved aging under directional selection either. Reduction of relatedness decreases the evolved level of aging; chance relatedness favors non-aging genotypes. The applicability of our results depends on empirical values of directional and kin selection in the wild.

**Conclusions:**

We found that aging can positively be selected for in a spatially explicit population model when sufficiently strong directional and kin selection prevail, even if reproduction is sexual. The view that there is a conceptual link between giving up clonal reproduction and evolving an aging genotype is supported by computational results.

**Supplementary Information:**

The online version contains supplementary material available at 10.1186/s12915-023-01716-w.

## Background

Aging (or senescence) is an increase in mortality and/or a decrease in fertility with age. Over a century ago, Weismann [1] suggested that death is a mechanism that had evolved through natural selection for removing enfeebled, older individuals and promoting the succession of generations. Besides this idea being a type of formerly discredited group selection mechanism seeking group-benefit adaptations (Weismann abandoned the idea and later on advocated non-adaptive concepts; [2, 3]), the argument was demolished by Medawar [4] as it already assumes what it sets out to conclude. Current thinking generally admits that in species capable of repeated breeding aging is a non-adaptive side effect of the weakening power of natural selection to maintain fitness at older ages [4–8]. This view has been disputed on several fronts. Demographers have undermined the claim that senescence is inevitable, both theoretically [9–11] and empirically [12–14], and several authors have recently turned to Weismann’s suggestion that programmed aging (i.e., that there are specific senescence genes), or the direct selection for a life end, can indeed evolve as an adaptation in its own right [15–19]. Contrarily to the claim by non-programmed aging theories ([20], p. 39) that “Death from senescence is itself in many species so rare an event in the wild state that failure to senesce early, or at all, has little value from the point of view of survival,” there is ample evidence that aging does happen in wild populations [21–26]. This is a critical ingredient to the revival of programmed aging theories because it means that natural selection might, in principle, act on aging.

A full understanding of the variety of aging patterns needs a broader theoretical perspective [11, 27] than that provided by classical evolutionary theory [28]. It is of note that classical theories of aging do not seem predict well the pattern of different aging dynamics in wild [29]. We now have numerous examples of evolutionarily conserved genes associated with aging [30]; however, the recurrent idea that aging can be programmed (apart from the case of semelparous organisms) or be adaptive per se still generates heated debate [31]. Perhaps most notable is Kowald and Kirkwood’s paper [32], in which they took seriously the theoretical possibility that aging could be selected for its own sake and critically reviewed various models trying to establish the case. After some cautious analyses, they concluded that none of the models considered stands close scrutiny, either because they are conceptually wrong, assume unrealistic high mutation rates, or the rules imposed in spatial simulations allow for the coevolution of programmed death. Kowald and Kirkwood’s [32] overall conclusion is that non-adaptive aging theories are still the best explanation for the evolution of the aging process. The only directly relevant paper missed by Kowald and Kirkwood [32] is that of Yang [33], which presents a metapopulation model assuming sexual reproduction (referring to potentially immortal individuals as selfish variants) with the finding that a lifespan (by “suicide”) readily evolves. We explain in the Discussion why we are dissatisfied with that model.

The intellectual stimulation for our present study comes primarily from a number of sources: (i) Weismann [1] argued for aging as “good for the species”; (ii) [34] argued that faster turnover of genes, aided by evolved aging, can be favored by kin selection; (iii) aging may be regarded as an evolvability component similar to genetic recombination in a spatially explicit, individual-based competition model under recurrent mutations for increased competitiveness [35]; and (iv) agers can oust non-agers in competition for an explicit resource [36]. But all these sources suffer from serious shortcomings, as explained by Kowald and Kirkwood [32]: (i) has been debunked by Medawar [4] and even Weismann himself [2, 3]; in (ii) lifespan reduction gives an evolutionary advantage and a (demographic) disadvantage, but benefit and cost are uncoupled so that one may arbitrarily make the former bigger than the latter; in (iii) selection for aging evaporates when the probabilistic rule for competition is slightly altered, or when reproduction is sexual, or when the mutation rate is reduced to realistic values; finally, (iv) gives no explanation for the putative advantage of aging that is found to be cryptic selection for mobility under the given rules of the spatial simulation. Importantly, in this paper, we mend the criticized defects of the previous modeling approaches.

After evaluating and criticizing Goldsmith’s [16] evolvability hypothesis for the adaptive evolution of aging, Kowald and Kirkwood [32] (p. 989) concluded that “Evolution is myopic such that there is no current reward for possible benefits in a far future.… In any case, a programmed limitation of the lifespan only has disadvantages (by killing agents) without any compensatory benefit.” In his response to Kowald and Kirkwood [32], Goldsmith [37] (see also [38]) insists on the increasing relevance of adaptive aging theories leading to an aging program that regulates lifespan in order to obtain an evolutionary benefit for a population of individuals that possess the program. He also criticizes Kowald and Kirkwood’s [32] analyses and claims that some of their assumptions are incorrect. However, it is still unclear whether aging can evolve assuming Goldsmith’s [16] evolvability hypothesis.

It is important to make a distinction between programmed aging in the narrow sense and adaptive aging in the broad sense [39], although (again) there are different views on this. One approach holds that programmed aging should entail a conserved pathway, potentially regulating many downstream genes, so that the process would be akin to normal development or apoptosis [29]. “Catastrophic” aging (as in semelparous organisms) can justifiably be regarded as programmed aging, a fact that tells us that aging can indeed be adaptive but does not imply that all adaptive aging is necessarily programmed in the described sense. We are interested in adaptive aging in the broad sense, in case of which selection would favor genetic variation that “lets deterioration go”—similar to what is envisaged by the disposable soma theory, but now for a different reason. We call this form “diffuse adaptive aging,” and we present an evolutionary mechanism for it, more robust (but still conditional) than ever before. Our present study grounds on a spatial, individual-based simulation model to give numerical support to the adaptive aging idea. The model assumes two ecological scenarios to which populations are allowed to adapt with their evolvable life history parameters (aging rate and fecundity): a directionally changing environment with fecundity as the target of selection as well as a constant stabilizing selection regime for comparative purposes.

Fecundity and aging rate are multilocus traits with binary allele sets randomly distributed on a single chromosome; each locus can toggle its allele from the “ON” to the “OFF” state or vice versa by mutation during reproduction. The fecundity ($\varphi$) of an individual depends in a multiplicative manner on the number of its fecundity alleles matching the actual optimum allelic pattern set by the periodically changing environment. This implements a sustained directional selection force on fecundity, while the stabilizing selection scenario keeps the optimum allelic pattern static. The actual death probability of an individual is the sum of a constant (age independent) background mortality (${\delta }_{0}$) and a linear age ($\tau$) dependent component ($\alpha \cdot \tau$) in which $\alpha$ is the evolvable rate of aging, the focal target of the evolution of senescence. Note that the background mortality is attributed to external (environmental) accidents independent of age: an individual with mortality ${\delta }_{0}$ is considered genetically immortal (“young forever”); therefore, mutations affecting aging rate cannot drive death probability below ${\delta }_{0}$. We assume asexual and sexual haploid populations of individual agents, allowing bit-flip type mutations with per bit probability ${p}_{f}$ and ${p}_{a}$ on their fecundity and aging loci, respectively. An empty site of the 2-dimensional lattice arena can be populated from its *n*-Moore neighborhood with chances of neighboring agents to win the empty site proportional to their actual fecundities. A complete asynchronous random updating cycle of the whole lattice defines a generation. At every $T$ th generation, the optimum of the environment (thus also the target phenotype) shifts by one element; *T* is referred to as the “fitness period dilution.” Time is measured in generations. For a complete specification of the algorithmic implementation of the simulation model, see the “Methods” section.

Table 1 summarizes the notation and the standard parameter values used in the simulations, although we have also tested the effect of changing relevant parameter values in the model (see below). Additional file 1: Fig. S1 is a schematic representation of the main events simulated in the model. All simulations were performed in C.

**Table 1:** Summary of notation and parameter values

Symbol	Meaning	Standard values
$N\cdot N$	Grid dimensions	$200\cdot 200$
${L}_{\mathrm{f}}$	Number of fecundity loci	50
${L}_{\mathrm{a}}$	Number of senescence loci	50
${\delta }_{0}$	Baseline mortality	0.05
${\tau }_{i}$	Actual age of the *i*th individual	
$\alpha$	Rate of aging	
$b$	Base of fitness for fecundity	1.2
$n$	Size of the Moore neighborhood	1
${s}_{i}[t]$	Number of matching fecundity alleles of the *i*th individual with the actual target phenotype at time *t*	
${\phi }_{k}[t]$	Target phenotype at time *t*	
$T$	Number of generations before a random element of the target phenotype is flipped (“fitness period dilution”)	10
${p}_{\mathrm{rec}}$	Probability of single point recombination	1
${p}_{\mathrm{f}}$	Mutation rate for fecundity (per bit flip probability of fecundity alleles)	0.01
${p}_{\mathrm{a}}$	Mutation rate for aging (per bit flip probability of aging alleles)	0.01
*D*	Diffusion parameter (average number of site swaps/time unit)	0

## Results

In this section, we show that aging can evolve under directional selection if, and only if, (a) the scale of temporal change in the relevant environmental conditions is not much longer than the lifespan of individuals, and (b) competitive interactions are played out between close neighbors (i.e., at high population viscosity).

### Spatial dynamics

**Fig. 1.**
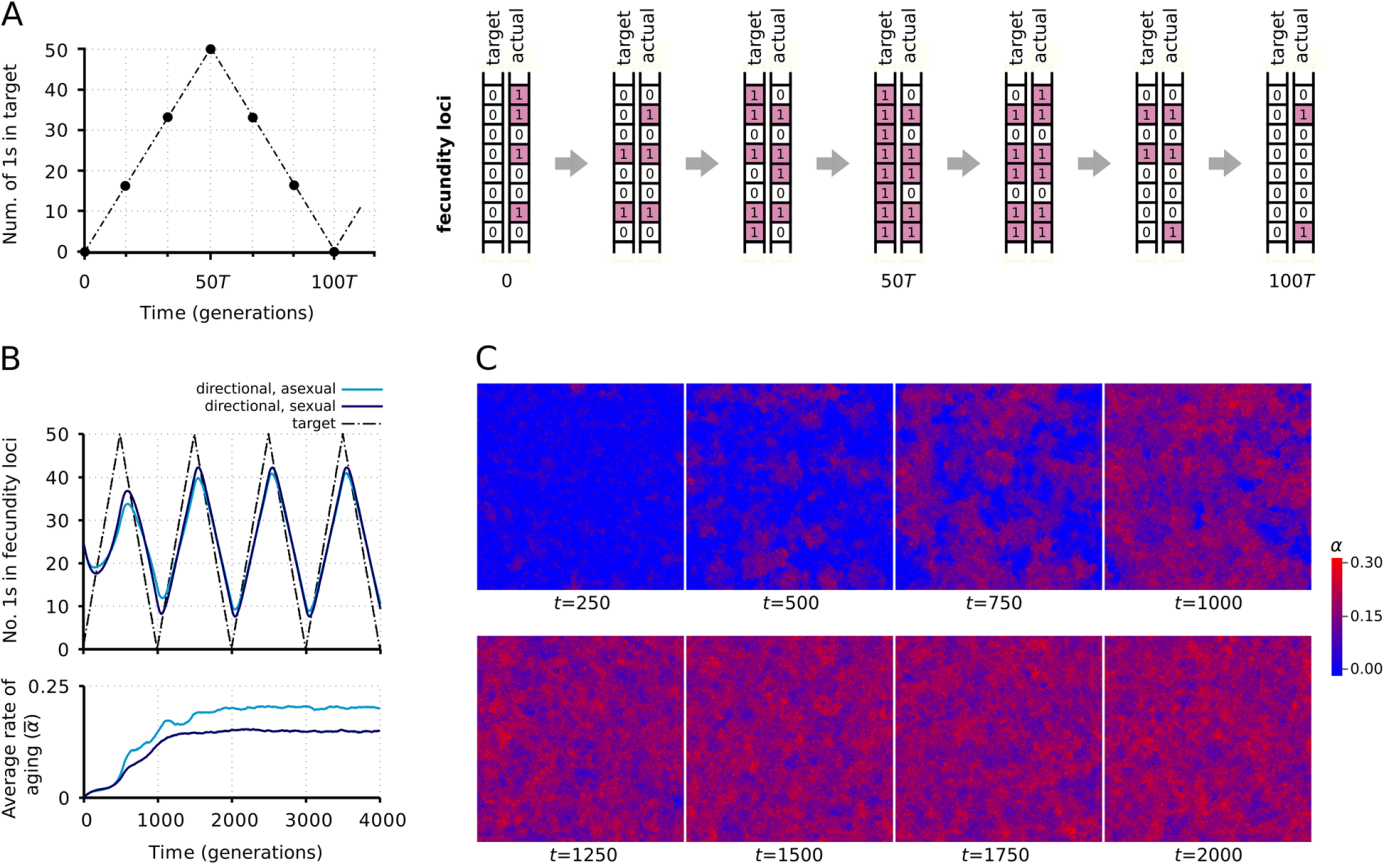
Evolutionary dynamics of aging. **A** one period of the fecundity target (left) and a possible realization with an actual series of genotype sequences tracing it by mutations (right), **B** the evolution of the average fecundity genotype toward the moving target through 4 target periods (4,000 generations) (upper plot) and the lattice average of the aging rate during these 4 target periods in the directional selection regime (lower plot), **C** consecutive snapshots of simulations with sexual reproduction. Top row: transient phase; bottom row: stationary phase. Parameter values as in Table 1

**Fig. 2.**
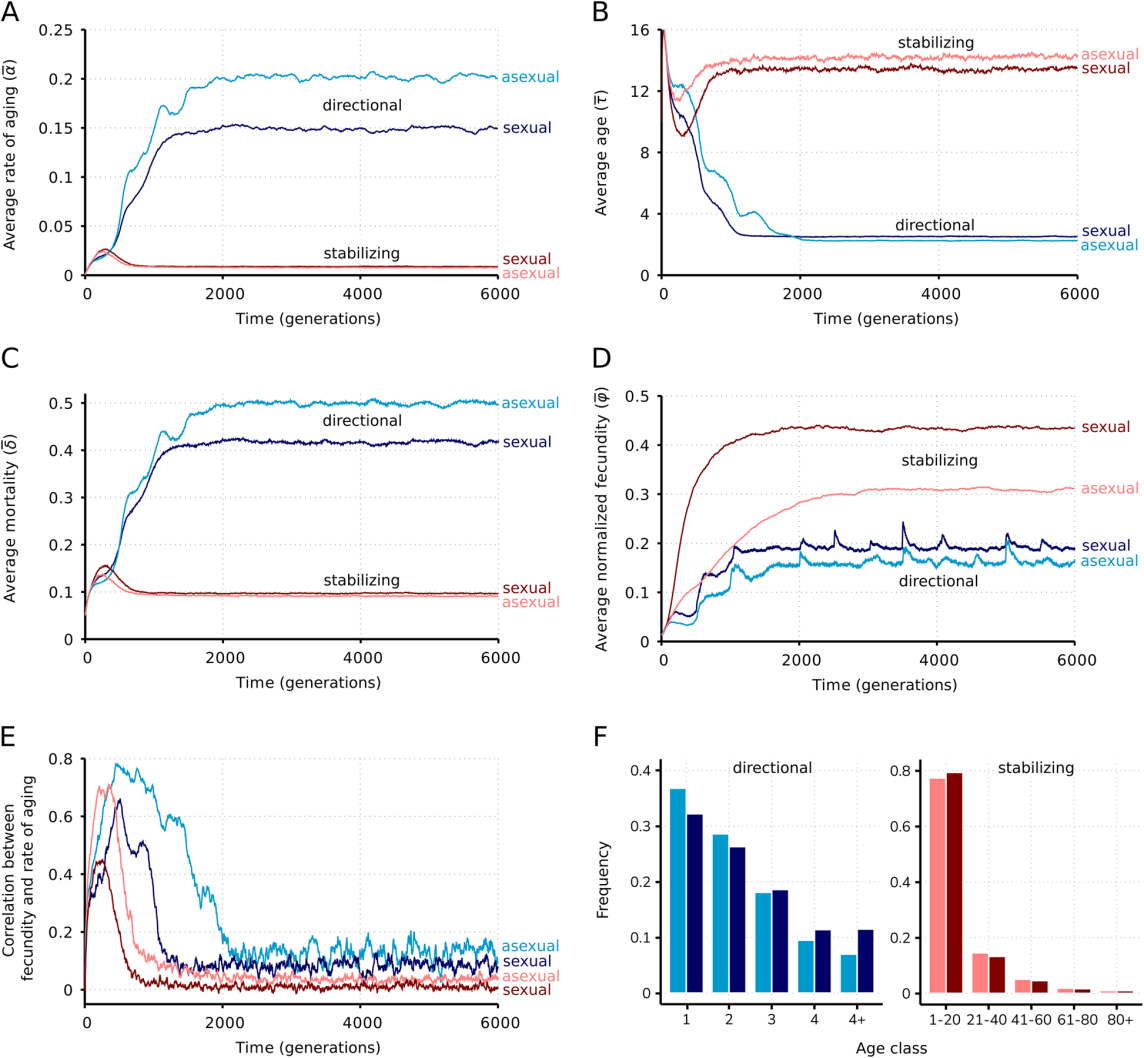
Quantitative aspects of evolving senescence across the model simulations. The lattice average of **A** rate of aging $(\overline{\alpha })$, **B** age of individuals $(\overline{\tau })$, **C** mortality $(\overline{\delta })$, **D** normalized fecundity $(\overline{\varphi })$, **E** Pearson correlations between fecundity and rate of aging as functions of time. **F** Age distribution of populations at $t=6000$ under directional selection (left panel) and stabilizing selection (right panel). Blue: directional selection; red: stabilizing selection. Darker shades represent sexual populations, lighter shades show the corresponding asexual cases. Parameters are as in Table 1

The model was run with the standard parameter values (Table 1) for 10,000 generations. The grid remained almost saturated with individuals (the average ratio of empty sites was less than ${10}^{-3}$). Figure 1A, B illustrate how the allele distribution of fecundity loci tracks the moving target set by permanent environmental change. Figure 1C shows that the system does not approach a stationary state on the local scale because an intransitive cycle of dominance and the “self-thinning” propensity of spatial domains of fast-aging individuals preclude its settling on a stable fixed point (this panel shows the case of sexual reproduction; the asexual case is similar). The dynamic spatial pattern of aging rate and fecundity is the result of a complicated local interplay of altruism (kin selection) and parasitism (cheating) resembling cyclical competition that changes spatial resolution as the system approaches its global steady state. The local altruists are faster aging individuals who sacrifice their longevity to benefit their more fecund kin neighbors, through more frequently leaving their own sites empty. The transient phase (up to about $t=2000$, Fig. 2) is characterized by a fast increase and then a similarly fast drop in the correlation of aging rate and fecundity (Fig. 2E), indicating the initial success of altruists and the subsequent invasion of cheaters. The latter are individuals of high fecundity, which do not maintain their high aging rate but occupy the sites left open by the altruists. Cheaters are kept in check by the moving target genotype in the directional selection regime, which does not apply in the stabilizing regime.

Recombination produces cheaters faster, which partially explains the lower average of aging rate (Fig. 2A) and the higher fecundity (Fig. 2D) in the global steady state ($t>2000$), along with the lower correlation between these variables in sexual populations (Fig. 2E). Note that, in contrast to several approaches in life history and aging theory, there is no genetic trade-off between fecundity and survival loci (hence fecundity evolves by directional selection only), and this is deliberate. It is consonant with the detected lack of correlation between aging rate and fecundity. Note that the antagonistic pleiotropy hypothesis assumes that such a correlation would build up in a constant environment [40], which would be detrimental in our case because the genotype that is fecund now is not fecund later.

The steady-state values of aging rate, fecundity, and their correlations are the lattice averages of the dynamical local equilibria between altruistic kin clusters and invading cheaters. The spatial distribution of $\alpha$ settles at a finite average domain size and maintains the coexistence of agers and non-agers on the spatial scale of the lattice. Movie files of the evolutionary dynamics for asexual (Additional file 2: Movie S1) and sexual (Additional file 3: Movie S2) populations can be downloaded from the Supporting Information.

### Lifespan and fecundity evolution under directional and stabilizing selection

The evolution of the relevant parameters, namely the extent to which the averages of rate of aging $(\overline{\alpha })$, age of individuals $(\overline{\tau })$, mortality $(\overline{\delta })$, and normalized fecundity $(\overline{\varphi })$ changed through time are plotted in Fig. 2 for both asexual and sexual reproduction and for both regimes of selection: directional and stabilizing.

The dynamical behavior of the system can be summarized as follows. The rate of aging increases when selection is directional (Fig. 2A), suggesting that individuals with shorter lifespans (Fig. 2B) can better adapt to the temporal change in the environment by producing fitter offspring. These individuals have, however, a demographic disadvantage due to higher mortality (Fig. 2C) entailing a lower realized fecundity (Fig. 2D) compared to their longer-lived counterparts (estimated as the difference in the equilibrium rate of mortality and fecundity between the directional and the stabilizing selection regimes). This occurs both in asexual and sexual populations, although some quantitative differences with respect to the mode of reproduction are clear from the simulations.

The reason why asexual populations reached a higher rate of aging (Fig. 2A) and mortality (Fig. 2C) under directional selection than sexual populations is as follows. Since asexuals by definition cannot recombine, but they can age, directional selection favors a higher degree of aging as a means to ensure faster production of stochastically lost or novel mutants per unit time (c.f., Fig. 2A, D; see the “Discussion” section for further analysis on the relation of mutations, recombination and aging) (the periodic peaks in the fecundity (Fig. 1B) are the consequence of the implementation of the directional selection: the slowly adapted (delayed) fraction of the population has higher fecundity right after the turning point—at $k\cdot {L}_{\mathrm{f}}\cdot T$, where $k$ is a positive integer—of the directional target).

**Fig. 3.**
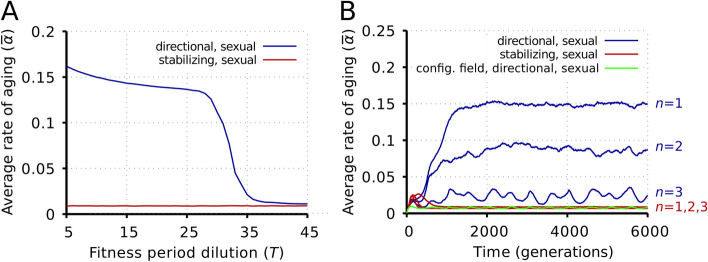
Effects of fitness epoch length and local neighborhood radius on the rate of aging. **A** Evolution of the lattice average of rate of aging ($$\overline{\alpha }$$) as a function of the fitness period dilution ($$T$$). Average of 100 runs; standard deviations within the range where aging can evolve are negligibly small (on the order of $${10}^{-3})$$. Other parameters are as in Table 1. **B** The lattice average of rate of aging ($$\overline{\alpha }$$) as a function of time for different neighborhood radii ($$n$$) assuming a sexual population. Note that the value of *n* is inconsequential under the stabilizing selection regime. Other parameters are as in Table 1

Under directional selection, the average lifespan evolved from an initial value of approximately 12 to a stable final value of approximately 2.5 (Fig. 2B). In other words, at the beginning, the average lifespan was slightly higher than the $T=10$ characteristic time of optimal fitness change used in the simulations (Table 1), and at the end, this change was 4 times the lifespan of individuals. These results seem to be at odds with Kowald and Kirkwood’s ([32], p. 989; emphasis added) claim that “If a change in the environment (here a switch of the direction of selection) happens on a *much longer time scale* than the lifespan of individuals, there is no selection pressure to prepare for such a distant event. And if the environment changes on a time scale that is comparable to the species lifespan, then not enough time has passed to diminish the genetic variation of the population.” Lifespan continued to evolve in our model when lifespan was similar to $T$, thus invalidating the second part of their claim. But the question still remains: how long is a *much longer time scale*? We have found that there is a constraint on the half-period $\left(T\cdot {L}_{\mathrm{f}}\right)$ of the target genotype: aging does not evolve when $T\approx 32$ or higher (Fig. 3A). Therefore, we conclude that Kowald and Kirkwood’s [32] were partially right: swift environmental changes do select for a fast rate of aging, but slow environmental changes in relation to the lifespan of individuals preclude the evolution of aging in our model.

### Kin selection

The evolution of the rate of aging requires spatial (neighborhood) interactions that render kin selection effective in our model. Increasing the neighborhood radius decreases the average level of steady-state rate of aging, whereas increasing its fluctuations through increasing mixing weakens kin selection. Figure 3B shows the effect of increasing the neighborhood radius on the rate of aging. In the configuration field limit (where the potential parents are chosen from eight random lattice sites and are, therefore, unrelated), aging cannot evolve: the equilibrium rate of aging is the same as in the case of stabilizing selection.

The kin selection effect is realized through a peculiar spatial mechanism that builds local gradients, allowing immediate (kin) neighbors to be only slightly different in their rates of aging and thus their competition for empty sites to be minimized. Additional file 1: Fig. S2A demonstrates the existence of such local gradients: neighbors of increasing distances apart differ more in aging rate in the statistical sense until the mean length of the local gradients is exceeded, where the plots saturate. Additional file 1: Fig. S2B also shows that the aging rates of individuals six sites apart differ more on average than those of immediate neighbors (one site apart), but the range of the latter is conspicuously wider. We attribute this effect to the fact that many of the immediate neighbors are located on patch borders where high-aging-rate individuals encounter low-aging-rate individuals. On these borders, the neighbors are either very different in terms of aging rate (no competitive exclusion of agers by non-agers yet) or they are very similar, after the exclusion has taken place. The spatial dynamics of such gradient patches (i.e., the permanent re-invasion of expanding ager patches by non-agers from the periphery) keep the spatial pattern of the lattice in a fluid motion.

### Parameter screening

Changing parameter values remained inconsequential on the proportion of occupied sites in the grid, which always remained higher than 0.999. However, the actual parameter values do affect the dynamically relevant demographic features of the model.

#### Mutation

We have tested the effect of changing the mutation rate (per bit flip probability) in the range $5\cdot {10}^{-4}\le p\le 0.025$ (Supporting Material). The results can be summarized as follows.

Fecundity (Additional file 1: Fig. S3D) decreases with increasing mutation rate (*p*) in the stabilizing selection regime because of the higher mutational load. With directional selection, the population cannot adapt to the changing target genotype when $p<0.006$ neither can the rate of aging evolve below this mutation rate (Additional file 1: Fig. S3A). The “beneficial” (sensu the evolvability hypothesis) rate of aging occurs at intermediate mutation rates ($0.006<p<0.013$). This “beneficial” effect can be estimated as the difference in the equilibrium rate of aging between the directional and the stabilizing selection regimes (at high mutation rates, at which this difference vanishes, aging is increasing, but not because of its beneficial effect through providing adaptability in fecundity; it is the simple consequence of the increasing mutational load).

The evolution of lifespan (Additional file 1: Fig. S3B) is in line with the evolution of the rate of aging. Mortality (Additional file 1: Fig. S3C) is a composite measure of individuals’ age and the rate of aging (Eq. 1), and its qualitative behavior also follows the trends observed for the rate of aging.

A superimposed plot of the average rate of aging $(\overline{\alpha })$ and the average Hamming distance from the target genotype $\left(\overline{s }\right)$ as functions of the mutation rate ($p$) can be seen in Additional file 1: Fig. S4A (stabilizing) and Additional file 1: Fig. S4B (directional) (Hamming distance counts the number of different loci between two sequences). In the stabilizing regime, $\widetilde{s}$ increases with small but increasing mutation rate, while the aging rate remains low. At a critically high mutation rate of $p\approx 0.012$ for sexuals and $p\approx 0.019$ for asexuals, there is a sharp increase in $\overline{\alpha }$.

#### Recombination

Under directional selection, the rate of aging is decreasing with increasing recombination rate (Additional file 1: Fig. S5A). This is due to the potential of recombination to produce good fecundity genomes (ones fitting to the actual target pattern) faster than mutation alone could, and these better genomes will be selected for and spread in the population (see Additional file 1: Fig. S5B), an option not given to asexuals. In these finite populations, recombination mitigates the Hill-Robertson effect by increasing the fixation of favorable mutants and expediting selective sweeps [41].

### Invasibility analysis

We have tested the invasion of individuals with a very low rate of aging $(\alpha =0.01)$ into an established population of non-aging individuals for stabilizing selection without the rate of aging mutated, ${p}_{\mathrm{a}}=0$ (i.e., all individuals in the established population experience the age-independent baseline environmental mortality ${\delta }_{0}$). Invasion is not possible at any density of aging individuals. The explanation is straightforward: aging individuals suffer the demographic disadvantage of their shorter lifespan that cannot be counterbalanced by higher adaptability as the regime is stabilizing. Consequently, the small equilibrium rate of aging under stabilizing selection (cf. Fig. 2A) is due to a mutation-selection balance.

We have also investigated the invasion of individuals with no aging ($\mathrm{\alpha }=0$) into an established population of individuals with evolved aging under directional selection. We found that the aging rate of the invaders increases to the equilibrium level of the resident population: the system relaxes to its original state, even when the concentration of the invaders is very high (e.g., half of the population).

### The effect of baseline (extrinsic) mortality

It is a common (but theoretically questionable: [42]) prediction that the rate of aging will be higher with increased extrinsic mortality [4, 40], for which there is comparative evidence [43], but not without counterexample [44]. In our framework, the simplest demonstration of this effect is when the fertility loci are monomorphic (hence they cannot evolve) and only the alleles for the aging rate undergo evolution. Additional file 1: Fig. S6A shows the results for asexual and sexual populations. There is a steep rise in the evolved rate of aging with extrinsic mortality rate in both cases and the sexual population evolves a higher aging rate up to a critical extrinsic mortality rate, beyond which the asexual population takes over. Ultimately, all the alleles for aging go to fixation and the aging rate is maximized. Note that in order to maintain comparability with the directional selection model, alleles for the rate of aging have identical rates for forward and back mutations. Since there are recurrent mutations to aging, the establishment of a mutation-selection balance would be compelling even in an infinitely large population, effectively a quasipecies model in which the deleterious mutations produce the cloud around the wildtype [45].

The results are best interpreted in comparison to finite [46] and spatially resolved [47], stochastic quasispecies models with the added complication that those models did not consider either extrinsic or evolved mortality. In our model, extrinsic mortality liberates spatial sites and thereby decreases competition between demographically different genomes so that selection becomes less and less effective against higher levels of evolved aging. The sharp transitions indicate the location of the error threshold in the simulated populations. When the mutation rate is high (Additional file 1: Fig. S6A), recombination merely helps the spread of deleterious alleles (for higher aging). These findings are entirely novel, since they show that the correlation between aging and extrinsic mortality can naturally arise as a result of mutation and selection in a finite, spatially explicit setting in a constant environment.

When fertility loci are allowed to evolve, increasing baseline mortality has a weak but obvious enhancing effect on aging rate in case of directional selection also. The rate of aging increases sharply above a critical extrinsic mortality under the stabilizing regime (Additional file 1: Fig. S6B)—a finding grossly in line with the original prediction similar to the previous case (Additional file 1: Fig. S6A) when fertility loci did not evolve. For sexual populations (Additional file 1: Fig. S6B), the pattern is similar, but again in line with the previous model (Additional file 1: Fig. S6A). These graphs do not contradict the established wisdom per se, but they shed new light on the quantitative aspect of the prediction that warrants further scrutiny (cf. [7]).

To separate the effects of evolving the two mortality components (baseline and age-dependent), we have also tested the case with age-independent but evolvable baseline mortality ($\alpha =0$). Baseline mortality was defined by the ${L}_{\mathrm{a}}$ senescence alleles in a way similar to the original model. The baseline mortality of individual $i$ was computed as: ${\delta }_{oi} =0.002+0.01\cdot \sum_{j=1}^{{L}_{\mathrm{a}}}{a}_{i,j}$, where the small prefactor 0.002 excludes infinite longevity in case of all-zero mortality alleles, and *a*_*ij*_ is the on(1)/off(0) index variable of mortality allele *j* of individual *i*. The process of recombination and mutation, and all other parameters of the model remain unchanged. We have measured the instantaneous death rates (cf. Equation (1)) as a function of the mutation rates; see Additional File 1: Fig. S7 for the average death rates of the original model for reference.

Our simulations with the modified model emphatically support the hypothesis that it is the age dependence of the individuals’ death probability that maintains an increased population average of mortality under directional selection. The evolutionary advantage of this increased mortality is through the gain in adaptability: the efficient differential elimination of “ecologically outdated” older individuals to make space for better-adapted young ones.

### Aging under directional selection is due to its indirect effect on evolvability

One might raise the objection that the increased rate of aging in sexual populations under directional selection could be a by-product of the increased mortality conferred on the population, simply because of directional selection itself acting in effect similarly to an increased extrinsic mortality rate. This is not so: Additional file 1: Fig. S8A clearly shows that when the rate of aging is fixed at various constant values, the tracking of the moving target is better with a higher rate of aging. Also, the simulation experiment with age-independent, mutable baseline mortality (Additional file 1: Fig. S7) confirms that age-independent mortality cannot evolve above its mutation-selection equilibrium even under directional selection.

### The effect of spatial mixing

The mixing effect is due to two different mechanisms: offspring moving away from the parent to a maximum of *n* sites and diffusion by site swaps. These have been studied separately and shown in Fig. 3B and Additional file 1: Fig. S9, respectively. Not surprisingly, both mixing mechanisms decrease population viscosity and thus diminish the positive effect of kin selection on the evolution on aging.

## Discussion

Weismann [1] was the first scientist trying to explain the usefulness of death in evolution and endorsed the idea known today as programmed aging. We show here that adaptive aging can evolve, and is maintained by kin selection (rather than group selection as in [48]), as long as selection is persistently directional and the scale of temporal change is not much longer than the lifespan of individuals. Therefore, our results question the claim by Kowald and Kirkwood [32] that aging does not evolve according to the evolvability hypothesis [16] but also put constraints on its general validity. We emphasize that (i) the demographic cost of aging is automatically effective in the model, (ii) directional selection acts in a general way, thus allowing several concrete realizations (including host-parasite dynamics, for example), (iii) mutation rates are not exaggerated, and, very importantly, (iv) non-aging genomes as “parasites” on agers cannot unconditionally halt the establishment of senescence in sexual population.

Note that with directional selection acting on survival (instead of fecundity) the direct and the indirect effects on mortality would counteract, thus weakening and possibly canceling each other because selection for increased net fitness would drive the average death rate down, whereas maintaining adaptability under directional selection requires it to increase. Therefore, the fitness component to be directly targeted by directional selection must be fecundity that can evolve independently of mortality.

The only seeming precedent to our analysis is the paper by Yang [33]. Although it is also a metapopulation model (as ours) with sexual reproduction, and it also predicts evolved aging, there are important differences. The two most consequential of these are that—besides directional selection acting on fecundity—Yang’s model assumes individual experience (i.e., learning) to play a decisive role in determining individual fitness and builds on an implicit survival/fecundity tradeoff. Without these, the Yang model cannot evolve aging, probably due to another significant difference: the sublinear fitness function it uses, as opposed to our multiplicative one, which enables evolved aging without acquired “abilities.”

We emphasize that the spread of aging is always helped by increasing extrinsic mortality, consistent with observations, and in contrast to the finding that extrinsic and evolved mortality would be negatively correlated [35]. The rational for the latter finding was that the two sources of mortality would complement each other. We do not find numerical support for the claim and we can explain our contrary result. One should note that the original Williams argument [40] for the correlation of extrinsic and intrinsic mortality seems to be wrong, essentially because age-independent mortality cannot modify selection pressures on life history traits; only age-specific mortality can [42]. This fact also invalidates a potential counterargument that an *evolved but constant* mortality would be favored over evolved aging: if this were the case then a higher extrinsic mortality in our model would disfavor rather than promote evolved aging. Directional selection ensures just the latter: it affects, on average, more the older genotypes than the young ones by selective deaths, so our finding agrees with established theory. More surprising is the finding that that even a monomorphic population with fixed fertility can accumulate genes for faster aging, positively affected by extrinsic mortality. Why so? This happens when mutations recurrently produce aging genotypes. Even if only harmful, average aging will be nonzero because of mutation-selection balance even in an infinite population. In a finite population the accumulation of alleles for aging is faster through drift, the latter being enhanced by stochastic extrinsic mortality. Importantly, our results suggest reanalysis of quasispecies models in which death rather than replication rates are the target of selection. In a spatially resolved setting extrinsic mortality always creates free space, thereby relaxing selection. This effect is lacking in models considering differential replication only.

If we accept that a key feature of sexual recombination is a better response than that by asexuals to directional selection (e.g., [49, 50]), then the prevalence of sex indicates that directional selection is common, so the demonstration that evolution of aging can rest on directional selection can be considered significant. Our result that directional selection favors aging differs from the conclusion based on a refinement of the life-history approach [51] showing that senescence is the only stable solution (without tradeoffs), because the latter result assumes a constant environment—but remember that moderate aging evolves without directional selection in our model also.

It is remarkable that aging can evolve in a sexual population despite the fact that there is a kind of cheater problem because non-aging individuals can steal good genes for their offspring from aging ones, while not paying the demographic cost (cf. [32]). Note that in this sense, aging individuals are strong altruists that are favored by kin selection for their establishment in the population [52, 53]—exactly as we have found. A recent paper arguing in favor of evolvability also neglects the problem of sex [51].

A forerunner to our study is the uncited(!) paper by Eshel [54] investigating the evolution of aging and sexual and asexual reproduction in a setting of continuous directional selection due to host-parasite antagonism. Although he misses the cheater problem (stealing of good genes from agers by non-agers through mating with them), Eshel ([54], p. 34) makes a remarkable observation: “it is an undisputable fact that under a quite general condition, the process of natural selection does enable either full or partial sexual reproduction. This means that under these conditions, there must be some selective advantage to an investment in a sexually reproduced offspring over a similar investment in an asexually produced offspring, fully identical to the parent. Thus, it may not be surprising that, under the same environmental conditions, investment in a sexually produced offspring is, all the same, preferred by natural selection over investment in the preservation of one’s own organism. Indeed, for the dynamics of the population, preservation of one’s own reproductive activity for one more generation is equivalent to the reproduction of a new, genetically identical organism, which is to last for exactly one generation. It is therefore, suggested that the evolution and molding of senescence should be re-examined in connection with the evolution of sexual reproduction.” In a framework more phenomenological than ours, he shows that aging can be advantageous over asexual reproduction and that there are “non-Hamiltonian” regions of the parameter space in which aging is selected for reasons of, essentially, evolvability.

Our results indicate that more sex entails less aging in the present scenario—i.e., under directional selection a higher level of fixed recombination rate entails a slower evolved aging rate (Additional file 1: Fig. S5A). Conversely, a higher fixed aging rate entails a lower evolved recombination rate (the effect being more pronounced under directional selection: Additional file 1: Fig. S8B). This is a remarkable finding that touches on the famous problem of sex and death (cf. [55]) and fits Eshel’s insight that both sex and senescence help the replacement of outdated genotypes. However, to our knowledge, data are scarce on this point. It was found that excess chiasma frequency correlates with age to maturity in mammals, which favors the Red Queen hypothesis for sex [56]. It is also true that age at maturity is a good predictor of lifespan in birds and mammals [57, 58], which suggests a correlation between recombination rate and longevity—in other words more sex goes with less aging—again as we have found. There are indications that linkage equilibrium breaks down relatively fast in trees [59] that are plants with high longevity. A caveat is that we do not know enough about the strength of kin selection for most species.

It is very important that in our model processes that reduce the efficacy of kin selection reduce the chance of the establishment of aging. We considered two such effects: increased neighborhood size (Fig. 3B) and enhanced diffusion rate (Additional file 1: Fig. S9), both of which reduce the average rate of aging steeply through weakening kin selection. Kowald and Kirkwood (2016) attribute the spread of aging in the spatial model of Werfel et al. (2015) partly to the fact that local rules in that model establish correlation between aging and mobility, the latter being advantageous by providing (literally) an escape route from competition in physical space. We differ from that criticized model because (i) we have only one species, (ii) the escape route is in genotype rather than physical space, and (iii) kin selection is beyond doubt operational. The reason why aging did not evolve in Kowald and Kirkwood’s [32] simulations implementing Goldsmith’s [16] verbal arguments was because agents were allowed to move into free neighboring locations in the grid or exchange positions with neighboring agents. This undoubtedly lessens the strength of kin selection, which, as we have found, is a critical ingredient for aging to evolve. In his rebuttal to Kowald and Kirkwood [32], Goldsmith [37] apparently failed to appreciate this point because he seems to believe that population structure is not a requirement for the evolvability hypothesis of aging—contrarily to Mitteldorf and Martins ([35], p. 292), who write: “Aging cannot evolve in a panmictic population, and population viscosity is crucial to the effect that we model.”—which is probably the reason why Kowald and Kirkwood [32] implemented the movement rule in their simulations. For instance, in his book on the evolution of aging, Goldsmith ([60], p. 133; our addition in brackets) writes: “The mechanism suggested above [the evolvability hypothesis] therefore does not appear to require a ‘group’ of a size larger than that required for generic natural selection. Also, the effect of such an amplifying trait is very immediate, (one generation) and therefore the benefit is not delayed from or slower than the effect of the individual disadvantage, a perceived problem with group selection.” As far as we can tell, this reasoning is incorrect.

From the modeling point of view an obvious extension of the present work would be putting it into a haystack-like [61] multigenerational model [62] since this approach allows selection for strong altruism, as required for our dynamics, even in randomly formed groups [63], which can be expected to broaden the applicability of the “senescence as an evolvability component” idea. A further task is to analyze in more depth the possible epistatic interactions between loci affecting aging—theoretical models have the advantage that they can be defined as having no pleiotropic effects. This is important because epistasis has a profound effect on fitness landscapes [64].

In sum, we predict that *other things being equal*, directional selection (in a viscous population) can favor aging both in sexual and asexual populations and that recombination and aging rates are negatively correlated. The snag is, of course, that so many things may *not* be equal and this calls for close scrutiny in the future. Another prediction from this mechanism, if it works, is that there will be genetic polymorphism for lifespan, with a certain frequency of genotypes with modest rates of aging.

Bourke [65] considered possible links between aging and kin selection in a 2 × 2 table, where the lifespan of the focal individual may go up or down, and the recipient’s fitness in turn may be increased or decreased. The results presented here give an example for the case of a decreased lifespan of the donor increasing the fitness of the recipient. He concludes (p. 103) that “systematically applying kin selection theory to the analysis of the evolution of aging adds considerably to our general understanding of aging.” We concur by strengthening numerical support for the link between aging and kin selection. In fact, Ronce and Promislow [66] have shown that kin competition can reduce the strength of selection on survival and that mutations increasing mortality in some age classes can be positively selected for.

Finally, it is critical to clarify what we have and have not shown here. We have provided some numerical support to the idea that the benefit of programmed aging to the evolution process can offset its individual disadvantage. We have also shown that aging can evolve in an initial population of non-aging genotypes, which overcomes the criticism in Kowald and Kirkwood ([32], p. 990) that “Obviously, a theory that proposes that aging is genetically programmed has to explain how such a program can evolve from a nonaging state.” Also obviously, we have not shown that aging is programmed and simply call attention to some results in yeasts and worms [67–69] suggesting that there are mechanisms for limiting lifespan. As Kirkwood ([7], p. 437) aptly wrote: “if genes program aging, they do so only very loosely.” Our results are compatible with such diffuse adaptive aging, also supported by the inevitably emerging polymorphism in the aging rate. The latter should be studied also empirically in the future, if possible. In any case, as pointed out by Cohen et al. [31], a consensus among aging researchers on whether aging is programmed or not is unlikely to be arrived at soon [29].

## Conclusions

We have found that, to the contrary of previous objections, that aging can evolve as an evolvability trait in a spatially explicit population model. There are two conditions for this: sufficiently strong directional selection and a sufficiently high degree of relatedness (kin selection). Relatedness ensures that non-aging individuals cannot steel good genes from aging ones since these two types of individual mate less frequently than random. We find that a relatively small degree of aging also evolves under stabilizing selection but this is mainly due to mutation-selection balance and drift. In our model, recombination and aging play complementary roles since both help the establishment of newer, hence on average fitter, genotypes by mutation, recombination and selection. Emphatically, a higher extrinsic mortality does not annihilate selection for aging. The scope of these findings is an empirical question of the parameter values, especially those of directional selection and relatedness in natural populations. Limited available data are consistent with the feasibility of the presented model for senescence.

## Methods

As in [32], we used agent-based computer simulations with agents living on a $N\cdot N$ grid with periodic boundary conditions (a torus). Each site of the grid can be empty or occupied by an individual. The individuals are characterized by their genomes consisting of ${L}_{\mathrm{f}}$ fertility loci labeled ${f}_{k} \left(k=1,\dots ,{L}_{\mathrm{f}}\right)$, and ${L}_{\mathrm{a}}$ senescence loci ${a}_{j} \left(j=1,\dots ,{L}_{\mathrm{a}}\right)$ on a single chromosome. The positions of fertility and senescence loci are random and fixed through the simulation. We assumed binary loci. The dynamically relevant demographic features of the individuals are their death probability and fecundity, both of which have a genetic basis.

### Death rate

The instantaneous death rate (or hazard function) is the probability that an individual *i* dies during a time step (generation). It is an increasing function of the product of its senescence alleles and its age ${\tau }_{i}$ according to the function

1 $${\delta}_{i} = \left\{\begin{array}{ll}{\delta}_{0} +{\alpha}\cdot {\tau}_{i}, & \mathrm{if}\ \tau \le {\tau}_{\mathrm{max}}\\ 1, & \mathrm{if}\ \tau > {\tau}_{\text{max}}\end{array}\right.$$

where ${\delta }_{0}$ is the age-independent baseline probability of dying—i.e., similar to the “hazard factors” recounted by [70] that prevent potentially immortal individuals to live longer—and ${\alpha }_{i} =0.01\cdot \sum_{j=1}^{{L}_{\mathrm{a}}}{a}_{i,j} ({a}_{i,j}\in \{\mathrm{0,1}\})$ is the age-dependent mortality rate (hereafter referred to as “rate of aging”), which is an additive function of the senescence alleles in an individual’s genotype (note that the maximum value of $\alpha$ is $0.01{L}_{\mathrm{a}}$, ${\tau }_{\mathrm{max}}=\lfloor\frac{1-{\delta }_{0}}{\alpha }\rfloor$ is the maximum lifespan, $\lfloor.\rfloor$ is the floor function). Note that the random asynchronous updating algorithm—a necessary choice to approximate continuous time in discrete event simulations—allows the age of a few individuals to exceed ${\tau }_{\mathrm{max}}$. This rare occurrence does not affect the outcome qualitatively.

### Fecundity

The model implements persistent directional selection as a moving genomic target. The selective environment is represented by a time-dependent $\{\mathrm{0,1}\}$ membered ${\phi }_{k}[t]$ fecundity vector of length ${L}_{\mathrm{f}}$ ($k=\mathrm{1,2},\dots ,{L}_{\mathrm{f}}$), which determines the optimal genotype in the actual environment at time *t* (square brackets emphasize discrete time). The actual fecundity of individual *i* at time *t* increases with the similarity between the composition of its fecundity genome and that of the optimal genotype at that point *t* in time. Hence, the fecundity by definition is ${\varphi }_{i}={b}^{{s}_{i}}$, where $b$ is the base of fitness for fecundity and ${s}_{i} \left(0\le {s}_{i}\le {L}_{\mathrm{f}}\right)$ is the number of fecundity loci at which the alleles of individual *i* and that in the actual target genotype ${\phi }_{k}[t]$ are the same (multiplicative fitness). More formally, ${s}_{i}=\sum_{k=1}^{{L}_{\mathrm{f}}}{\delta }_{{f}_{i,k};{\phi }_{k}}$, where ${f}_{i,k}$ is the *k*th fecundity allele of individual *i*, and ${\delta }_{x;y}$ is the Kronecker-delta function: ${\delta }_{x;y}=1$ if $x=y$; ${\delta }_{x;y}=0$ otherwise. Note that the target fecundity genotype is the same for all individuals. Later on, we will normalize the fecundity to the $[\mathrm{0,1}]$ interval by dividing by the maximum attainable fecundity ${b}^{{L}_{\mathrm{f}}}$. Environmentally directed selection is maintained by periodical changes in the optimal fecundity vector ${\phi }_{k}={\phi }_{k}[t]$. Initially (at $t=0$), ${\phi }_{k}$ is a zero vector (i.e., alleles 0 increase fertility). After every $T$ time steps, one random zero element of the fecundity vector flips to the 1 state, and this continues until all values are 1 s. When this happens (after $T\cdot {L}_{\mathrm{f}}$ time steps), the same process is repeated in the reverse direction, i.e., after every $T$ time steps a single random element of the ${\phi }_{k}$ flips to zero. Therefore, the length of a whole period is $2\cdot T\cdot {L}_{\mathrm{f}}$. This choice of directional selection implements a continuous change in the target genotype, where the Hamming distance between two consecutive states is one. It also concurs with Kowald and Kirkwood [32] who also reversed the direction of selection at regular intervals.

We also used a stabilizing selection regime with the target genotype being a static (time-independent) constant vector consisting of random $\{\mathrm{0,1}\}$ elements: ${\phi }_{k}\left[t\right]={\phi }_{k}=Rnd\left(\mathrm{0,1}\right)$. This serves as a comparative situation to the sustained directional selection scenario, besides representing the effect of a static environment that has been suggested and evidenced as a likely ingredient contributing to the prolonged morphological stability and stasis of many species [71–75].

### Dynamics

Initially, all cells in the grid are occupied by individuals with ages ${\tau }_{i}$ drawn from a uniform distribution $U(\mathrm{0,1}/{\delta }_{0})$. Senescence alleles are set to the 0 state, and fecundity loci have random 0 or 1 values (this choice reduces the initial oscillations of the fecundity in the directional case; otherwise, it does not affect the outcome of the simulations). Therefore, at first, there was only an age-independent baseline mortality ${\delta }_{0}$, so we can test whether or not aging can evolve from a non-aging state [32].

The state of the population on the grid changes through an iteration of $N\cdot N$ Monte Carlo steps that defines a generation, followed by an age updating step at the end of the iteration. Each step starts by choosing a random target site from the lattice. If the site is occupied, the individual dies with probability ${\delta }_{i}$ according to Eq. 1. If the site is empty, it can be populated with the offspring of one of the surrounding individuals belonging to its *n*-Moore-neighborhood ($n=1$ corresponding to the standard 8-site neighborhood). Reproduction can be sexual or asexual. During reproduction, one parent is drawn from the neighboring individuals around the empty site with probability according to the relative fecundity weights of the parental candidates. If the chosen parent reproduces asexually, the offspring genotype will be a mutated copy of that parent. If it reproduces sexually, we choose (without replacement; i.e., we assume dioecy) a second parent from the remaining neighboring individuals with probability again corresponding to the relative fecundity weights. If there is no sexual pair of individuals in the neighborhood, then there is no sexual reproduction and the target site remains empty. We assumed single-point recombination with ${p}_{\mathrm{rec}}=1$, with the point of crossing over set at a random inter-locus position (so that at least one gene of the offspring comes from each parent), and also introduced mutations to the recombinant offspring chromosome. Mutations are bit-flip type gene mutations: ${f}_{i,k}^{\prime}={-f}_{i,k}+1$ and ${a}_{i,j}^{\prime}={-a}_{i,j}+1$, with per bit-flip probability ${p}_{\mathrm{f}}$ and ${p}_{\mathrm{a}}$, respectively. If not noted otherwise, the two mutation rates are assumed to be the same: ${p}_{\mathrm{f}}={p}_{\mathrm{a}}=p$.

### Spatial mixing

Besides the inevitable baseline individual mobility (which is due to progeny placed on empty sites at a maximum of *n* steps removed from the parent at each birth event), an additive, scalable component of mobility is introduced by allowing swaps between randomly chosen adjacent (orthogonal or diagonal) pairs of sites. *D* is the probability of such a swap occurring after each elementary birth/death update. Thus, $D=0.5$ amounts to a single step taken on the lattice, on average, by each site in a time unit.

### Supplementary Information

**Additional file 1:** **Supporting information. Fig. S1.** A series of idealized snapshots of some representative runs of the simulations. **Fig. S2.** Emergent patches of local gradients of aging rate in viscous populations. **Fig. S3.** The effect of the mutation rate on the behavior of the system. **Fig. S4.** The effect of mutation rate on aging. **Fig. S5.** The effect of the recombination rate on the behavior of the system. **Fig. S6.** The effect of baseline mortality on rate of aging. **Fig. S7.** The effect of evolvable baseline mortality without aging. **Fig. S8.** The effect of monomorphic senescence on fecundity and recombination rate. **Fig. S9.** The effect of mobility on aging.

**Additional file 2:** **Movie S1.** The time evolution of rate of aging (α) in the time interval 0≤t≤4,000. Reproduction is asexual, parameters as in Table 1.

**Additional file 3:** **Movie S2.** The time evolution of rate of aging (α) in the time interval 0≤t≤4,000. Reproduction is sexual, parameters as in Table 1.

## Data Availability

The datasets generated and/or analyzed during the current study and the source code are available in the GitHub repository, https://github.com/andszilagyi/AgeingEvol [76].
