# Supplementary material for: Directional selection coupled with kin selection favors the establishment of senescence
Source: BMC Biol. 2023 Oct 23;21:230. doi: 10.1186/s12915-023-01716-w (PMC10591417; doi:10.1186/s12915-023-01716-w)
Supplement: Supplementary file 1 — Additional file 1: Supporting information. Fig. S1. A series of idealized snapshots of some representative runs of the simulations. Fig. S2. Emergent patches of local gradients of aging rate in viscous populations. Fig. S3. The effect of the mutation rate on the behavior of the system. Fig. S4. The effect of mutation rate on aging. Fig. S5. The effect of the recombination rate on the behavior of the system. Fig. S6. The effect of baseline mortality on rate of aging. Fig. S7. The effect of evolvable baseline mortality without aging. Fig. S8. The effect of monomorphic senescence on fecundity and recombination rate. Fig. S9. The effect of mobility on aging. [file 12915_2023_1716_MOESM1_ESM.pdf]

Supplementary Materials for

Directional selection coupled with kin selection favors the establishment of senescence

András Szilágyi, Tamás Czárán, Mauro Santos, Eörs Szathmáry

This PDF file includes

Figs. S1 to S9:

- **Fig. S1** A series of idealized snapshots of some representative runs of the simulations.
- **Fig. S2** Emergent patches of local gradients of aging rate in viscous populations.
- **Fig. S3** The effect of the mutation rate on the behavior of the system.
- **Fig. S4** The effect of mutation rate on aging.
- **Fig. S5** The effect of the recombination rate on the behavior of the system.
- **Fig. S6** The effect of baseline mortality on rate of aging.
- **Fig. S7** The effect of evolvable baseline mortality without aging**.**
- **Fig. S8** The effect of monomorphic senescence on fecundity and recombination rate.
- **Fig. S9** The effect of mobility on aging.

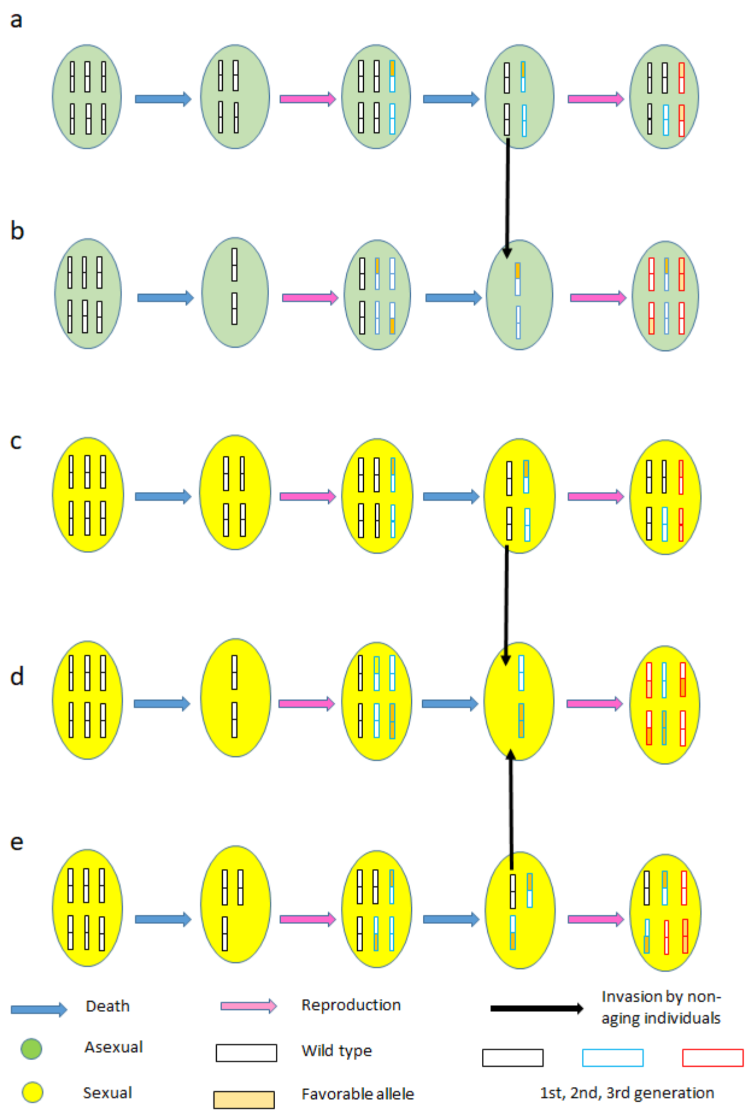

Fig. S1. A series of idealized snapshots of some representative runs of the simulations. Individuals mostly interact in local kin groups, with limited migration between them. Asexual and sexual populations are treated here separately. (a) and (c) assume low, while (b) and (d) assume low and high rates of aging. (e) assumes an intermediate value. The "place" of dead individuals is occupied by newborns from the next generation. Sexual populations evolve faster by recombination, hence they harbor a higher number of favorable alleles at the same epoch than asexuals, and there are more linked good alleles. The latter implies higher fertility = $(1 + s)^2$, modelled as higher chance of reproduction. Aging also speeds up evolution. When recombination and aging act together then a lower aging rate suffices, which explains that sexuals with an intermediate aging rate (e) can evolve at least as fast as asexuals with a higher aging rate (b). Local groups with a lower aging rate are more likely to penetrate those with a higher aging rate than vice versa.

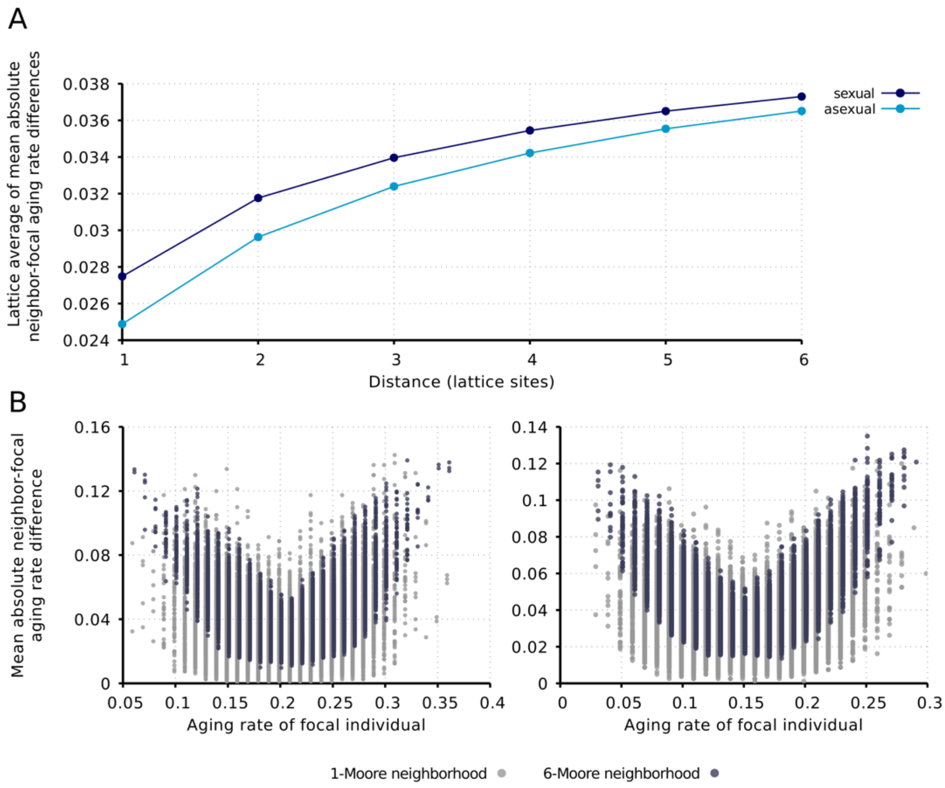

Fig. S2. Emergent patches of local gradients of aging rate in viscous populations. (A) plot of the lattice average of mean absolute difference in aging rate between close neighbors as a function of their distance (1-6). (B) The distribution of mean absolute differences in aging rates between close neighbors (of distances 1 and 6) as a function of the actual aging rate of the focal individuals. Notice the extended range for immediate neighbors (distance 1). Explanation in the main text.

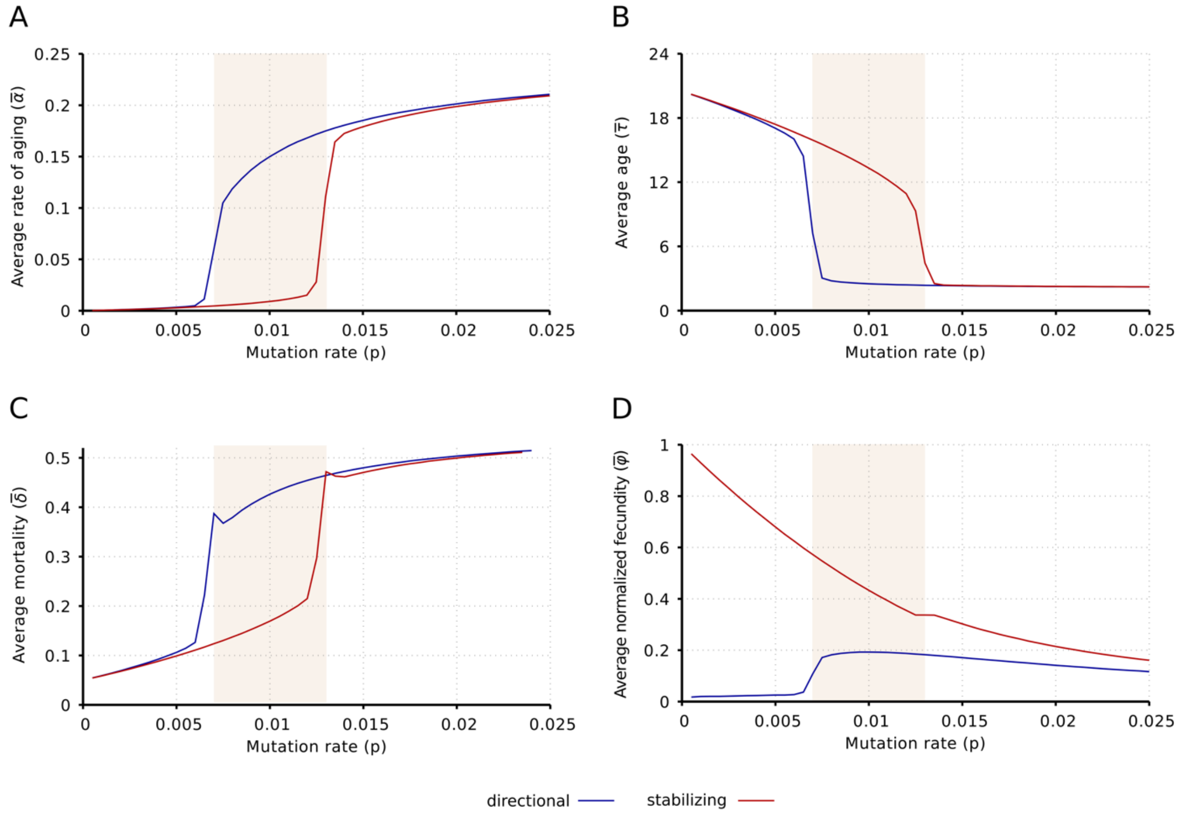

Fig. S3. The effect of the mutation rate on the behavior of the system in the $5 \cdot 10^{-4} \leq p \leq 0.025$ range. The panels show the lattice average of: (A) rate of aging ($\bar{\alpha}$), (B) age of individuals ($\bar{\tau}$), (C) mortality ($\bar{\delta}$), (D) normalized fecundity ($\bar{\varphi}$) as the function of the mutation rate. Blue: directional selection; red: stabilizing selection. Shading indicates the range of the mutation rate in which aging evolves. Other parameters are as in Table 1, the population consists of recombinant individuals only. The values are the averages of 100 runs, the standard deviations within the range where aging can evolve are negligibly small (in the order of 10^{-3}).

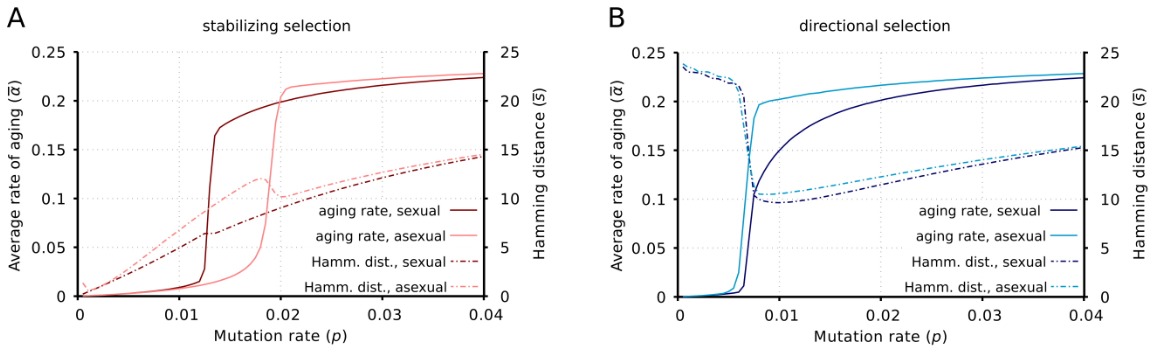

Fig. S4. The effect of mutation rate on aging. The lattice average of rate of aging ($\bar{\alpha}$) and average Hamming-distance of fecundity loci from the actual target ($\bar{s}$) as the function of the mutation rate p in (A) stabilizing selection regime, (B) directional selection regime. Other parameters are as in Table 1, the values are the averages of 100 runs, the standard deviations within the range where aging can evolve are negligibly small (in the order of 10^{-3}).

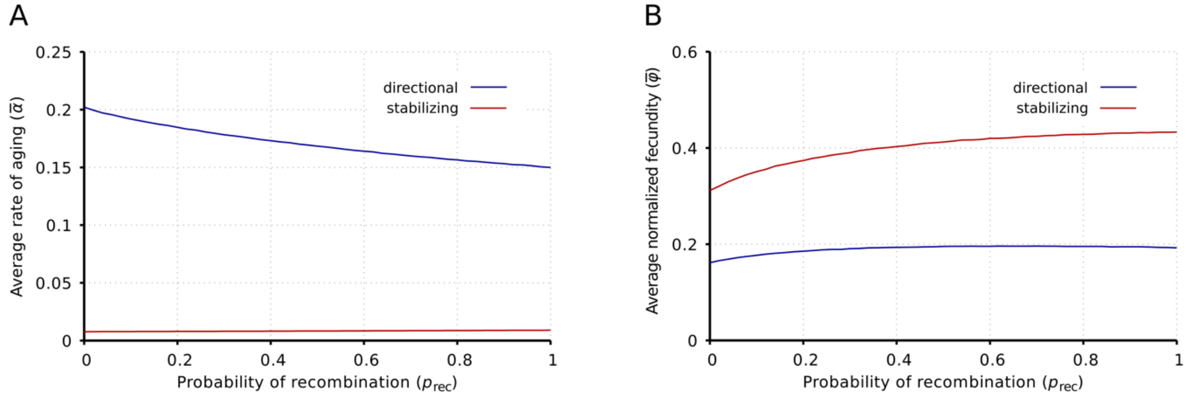

Fig. S5. The effect of the recombination rate on the behavior of the system. The panels show the lattice average of: (A) rate of aging ($\bar{\alpha}$), and (B) normalized fecundity ($\bar{\varphi}$) as the function of the mutation rate. Blue: directional selection; red: stabilizing selection. Other parameters are as in Table 1, the population consists of recombinant individuals only. The values are the averages of 100 runs, the standard deviations within the whole range are negligibly small (in the order of 10^{-3}).

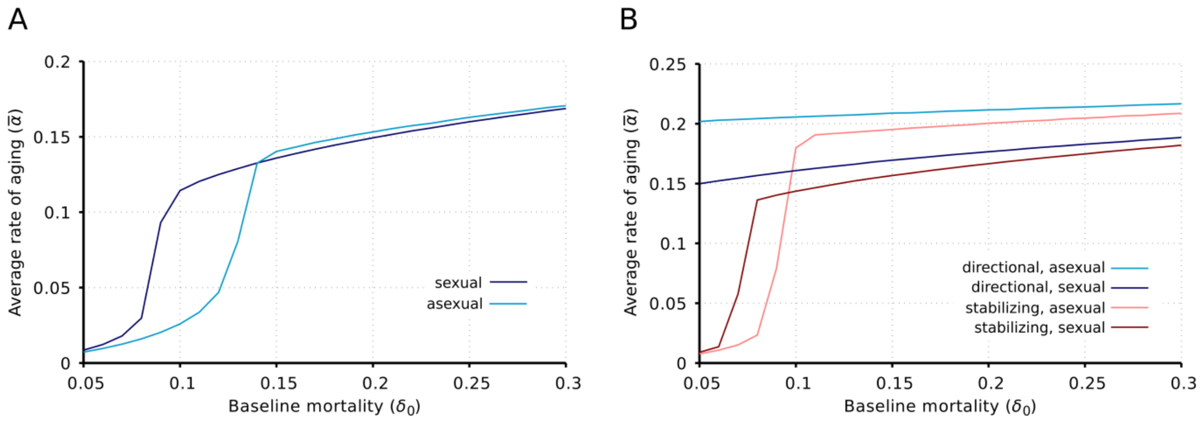

Fig. S6. The effect of baseline mortality on rate of aging. The lattice average of rate of aging ($\bar{\alpha}$) as the function of the baseline mortality (δ_0) (A) with monomorphic fertility loci for asexually and sexually reproducing populations, (B) in case of directional and stabilizing selection for asexually and sexually reproducing populations. Other parameters are as in Table 1, the values are the averages of 100 runs, the standard deviations within the range where aging can evolve are negligibly small (in the order of 10^{-3}).

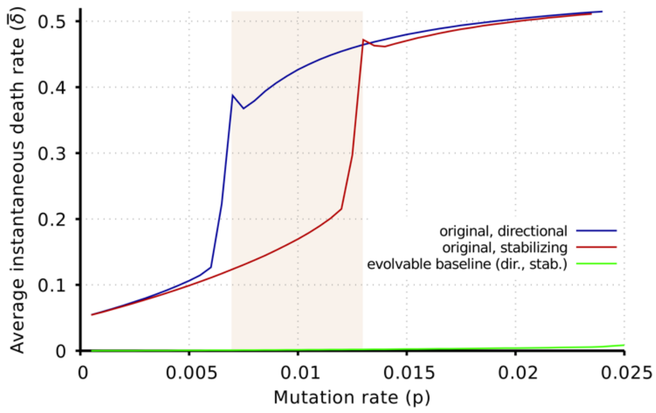

Fig. S7. The effect of evolvable baseline mortality without aging as the function of the mutation rate within the $5 \cdot 10^{-4} \leq p \leq 0.025$ range. The average instantaneous death rate (δ) without aging ($\alpha = 0$) (green curve, same for directional and stabilizing selection). The average instantaneous death rate in the original model under the stabilizing (red) and directional (blue) selection (the same as in Fig S2C).

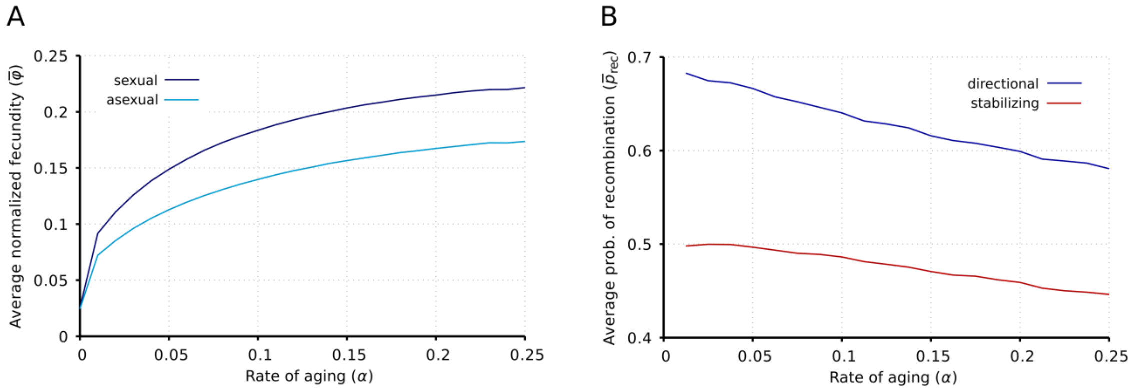

Fig. S8. The effect of monomorphic senescence on fecundity and recombination rate. (A) The lattice average of normalized fecundity ($\bar{\varphi}$) as the function of the rate of aging ($\bar{\alpha}$) with monomorphic senescence loci in case of directional selection for asexually and sexually reproducing populations. (B) The lattice average of the probability of recombination (p_{rec}) as a function of the rate of aging (α) with monomorphic senescence loci in case of directional and stabilizing selection. Other parameters are as in Table 1, the values are the average of 100 runs, the standard deviations within the whole range are negligibly small (in the order of 10^{-3}).

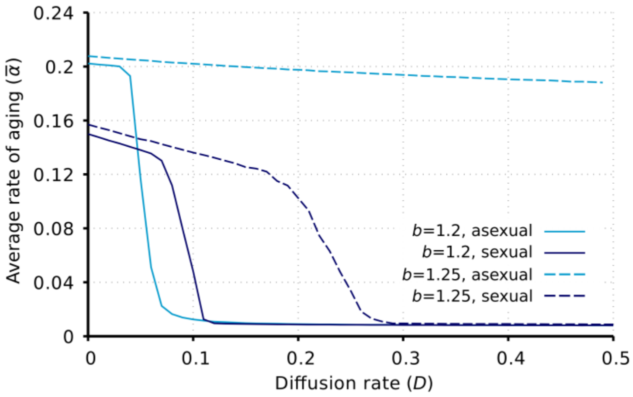

Fig. S9. The effect of mobility on aging. The lattice averages of the rate of aging ($\bar{\alpha}$) as functions of the diffusion rate, for directional selection with the standard ($b = 1.2$, continuous lines) and an elevated ($b = 1.25$, dashed lines) base of multiplicative fitness for asexually and sexually reproducing populations. Other parameters are as in Table 1; the values are the averages of 100 runs, the standard deviations within the range where aging can evolve are negligibly small (in the order of 10^{-3}).
